# Oral Metronomic Chemotherapy in Nasopharyngeal Carcinoma with Radiotherapy Interruptions: A Lesson Learned from the Lockdown Due to COVID-19

**DOI:** 10.2174/0115748928378983250604171225

**Published:** 2025-06-13

**Authors:** Yuan Zhou, Rui Zhou, Yi-Feng Yu, Zheng-Jie Huang, San-Gang Wu

**Affiliations:** 1 Department of Radiation Oncology, Xiamen Cancer Center, Xiamen Key Laboratory of Radiation Oncology, the First Affiliated Hospital of Xiamen University, School of Medicine, Xiamen University, Xiamen, 361003, People’s Republic of China;; 2 Department of Gastrointestinal Surgery, the First Affiliated Hospital of Xiamen University, School of Medicine, Xiamen University, Xiamen, 361003, People’s Republic of China

**Keywords:** Nasopharyngeal carcinoma, radiotherapy interruptions, metronomic chemotherapy, COVID-19, prognosis, compensatory treatment, S-1

## Abstract

**Purpose:**

Metronomic chemotherapy (MC) represents a therapeutic approach characterized by the long-term administration of chemotherapeutic agents at relatively low doses, with minimal or no drug-free intervals (US20150283237, CN111110681A). This study aimed to evaluate the treatment characteristics, prognosis, and efficacy of S-1 MC as a compensatory strategy for nasopharyngeal carcinoma (NPC) patients who experienced radiotherapy interruption (RI) during the COVID-19 pandemic.

**Methods:**

This study included NPC patients who experienced RI due to the COVID-19 pandemic. Patient characteristics, details of treatment after RI, compensatory treatment, and survival outcomes were analyzed.

**Results:**

A total of 8 patients were identified, with a median RI duration of 19 days. All patients received an additional fraction of radiotherapy due to the interruption. Following RI, all patients completed the recommended radiotherapy regimen and underwent comprehensive locoregional and systemic assessment three months post-treatment. Complete remission of the nasopharyngeal tumor and cervical lymph nodes was achieved in 7 (87.5%) patients. These patients were administered oral tegafur, gimeracil, and oteracil potassium (S-1) MC. All patients completed one year of MC without experiencing grade 3-4 adverse reactions. With a median follow-up of 34.4 months, no instances of disease recurrence were observed. The 2-year disease-free survival and overall survival were both 100%.

**Conclusion:**

MC may serve as an effective compensatory treatment strategy for NPC patients experiencing RI. These findings offer valuable insights for future clinical trials involving NPC patients with RI due to various reasons.

## 1 INTRODUCTION

Nasopharyngeal carcinoma (NPC) is a distinct subtype of head and neck cancer originating from the nasopharyngeal epithelium. While NPC is endemic in Southeast Asia, it is relatively rare in the United States or Europe [1]. NPC exhibits unique epidemiology patterns, biological behavior, treatment response, and prognosis compared to other head and neck cancers [2, 3]. Notably, NPC is highly sensitive to both chemotherapy and radiotherapy. In contemporary clinical practice, intensity-modulated radiation therapy (IMRT) is the primary radiation modality for NPC. With the advancement of comprehensive treatment strategies, the 5-year overall survival (OS) rate for NPC has reached approximately 85% [4, 5]. However, 10-30% of patients experienced radiotherapy interruption (RI) due to factors such as public holidays, machine malfunctions, acute toxicity, and patient compliance [6-8]. Importantly, RI has been significantly associated with poor prognosis in NPC patients [6-8]. Despite this, there remains no consensus on optimal compensatory treatment strategies for NPC patients experiencing RI.

The coronavirus disease 2019 (COVID-19) pandemic, which emerged in 2019, led to widespread lockdowns globally to curb disease transmission [9]. China, among other countries, faced significant disruption due to the pandemic [10]. Many cities and healthcare institutions implemented lockdowns, posing substantial challenges to cancer treatment [11-13]. The COVID-19 pandemic resulted in delayed diagnosis and treatments, which are known to increase cancer-related mortality [14-16]. Several studies have highlighted that the COVID-19 pandemic causes treatments to be delayed or interrupted for NPC patients, with significant implications for survival outcomes [15, 17]. Furthermore, the pandemic disrupted standard radiotherapy protocols due to resource reallocation, patient access issues, and infection control measures, raising concerns about their impact on patient prognosis. Deviations or delays in radiotherapy can compromise therapeutic efficacy, potentially leading to adverse outcomes. Therefore, exploring compensatory treatment strategies is critical to mitigate the negative effects of RI and maintain treatment efficacy. Compensatory treatment following RI involves the administration of specific therapeutic agents to mitigate the risk of tumor recurrence or enhance the efficacy of subsequent treatments. Although these agents may not represent novel patent-protected compounds, their application in this unique clinical context offers a valuable framework for optimizing the use of existing therapies in challenging scenarios. This approach not only addresses the immediate need to counteract the adverse effects of RI but also provides insights into the broader field of anti-cancer drug discovery. By refining the clinical application of approved drugs and generating novel clinical data, such strategies can inform future drug development and repurposing efforts, thereby advancing personalized and adaptive cancer treatment paradigms.

Metronomic chemotherapy (MC) is a therapeutic regimen involving the long-term administration of chemotherapeutic agents at relatively low doses, with minimal or no drug-free intervals (US20150283237, CN111110681A) [18, 19]. This approach minimizes side effects by utilizing doses significantly lower than the maximum tolerated dose (MTD) employed in traditional chemotherapy. Unlike traditional regimens, which are administered in limited cycles to avoid drug resistance and excessive organ damage, MC involves frequent, uninterrupted dosing. MC with S-1 (tegafur, gimeracil, and oteracil potassium) had demonstrated antitumor and anti-angiogenic effects with reduced toxicity, making it an effective treatment for various cancers [20-24]. In this study, we aimed to evaluate the treatment characteristics, prognosis, and efficacy of compensatory MC using S1 of NPC patients who experienced RI due to the COVID-19 pandemic at our institution.

## 2 MATERIALS AND METHODS

### 2.1 Patient Selection

This retrospective study included patients who experienced RI due to the COVID-19 pandemic between September 13, 2021, and October 8, 2021. Patients who met the following criteria were included in the analysis: 1) diagnosed with stage I-IVA NPC according to the eighth edition of the American Joint Committee on Cancer staging system; 2) experienced RI due to the COVID-19 pandemic; 3) completed the recommended radiotherapy dose following the interruption of IMRT; 4) possessed adequate haematologic, liver, and renal function to tolerate adjuvant MC. Patients were excluded if they had metastatic disease at the time of NPC diagnosis, had a history of previous malignancies, or were diagnosed with concurrent malignant diseases. This study was approved by the Ethics Committee of the First Affiliated Hospital of Xiamen University (approval number: XMYY-2024KY237), and informed consent was obtained from patients.

### 2.2 Measures

The following demographic and clinicopathological characteristics were included in the analyses: age, gender, histology, induction treatment before IMRT, concurrent treatment during IMRT, start and end dates of RI, treatment after RI, and treatment after IMRT. Plasma Epstein-Barr virus (EBV)-DNA load was measured before and after IMRT, as well as during follow-up.

### 2.3 IMRT and Chemotherapy

All patients received definitive IMRT. The prescription dose was 70 gray (Gy)/33 fractions for the primary gross tumor volume and involved cervical lymph nodes, 62 Gy/33 fractions for the high-risk clinical target volume, and 56 Gy/33 fractions for the low-risk clinical target volume. Institutional guidelines recommended IMRT alone for stage I disease, platinum-based concurrent chemoradiotherapy (CCRT) for stage II disease, and induction chemotherapy (IC) followed by platinum-based CCRT for stage III-IVa disease. For patients with locoregionally advanced NPC (LANPC) exhibiting T4 or N2-3 disease, one-year oral S-1 MC was recommended.

### 2.4 Retreatment after Radiotherapy Interruption

No standardized regimen exists for compensatory treatment following RT. In this study, patients who experienced RI during the COVID-19 pandemic received one additional IMRT fraction to address the biological dosimetry impact of RI. For patients who had not completed CCRT, the remaining cycles were administered. A previous study demonstrated that S1-based adjuvant MC significantly improves disease-free survival (DFS) and OS in high-risk NPC patients [25]. S-1 MC was implemented in our study.

### 2.5 Assessment of Adverse Events with S-1 Treatment

Adverse events associated with S-1 were monitored throughout S-1 treatment and classified according to the Common Terminology Criteria for Adverse Events (CTCAE), version 3.0. Blood tests and adverse event assessments were conducted biweekly.

### 2.6 Treatment Responses Assessment and Follow-up

Treatment responses were evaluated before IC and after IMRT using a nasopharyngoscope and magnetic resonance (MR) based on the Response Evaluation Criteria in Solid Tumors (RECIST). Patients were followed up every three months during the first three years, with clinical examinations, blood tests, EBV-DNA load measurements, nasopharyngoscopy, MR scan, chest computed tomography, and abdominal ultrasound performed. Bone scans were conducted only if clinical suspicion of bone metastasis arose. Patients with suspected disease recurrence underwent positron emission tomography/computed tomography (PET/CT) scans. The primary endpoints of this study were DFS and OS. DFS was measured from the day of the NPC diagnosis until disease recurrence and death from any cause. OS was measured from the day of the NPC diagnosis until death due to any cause.

### 2.7 Statistical Analysis

Categorical variables, including patient demographics, treatment characteristics, and treatment response, were summarized using frequencies and percentages. Continuous variables were expressed as median based on their distribution. Point estimates and 95% confidential intervals (CIs) were calculated using the Clopper-Pearson method. Survival probabilities for DFS and OS were estimated using the Kaplan-Meier method. All statistical analyses were performed using SPSS version 27.0 (SPSS Inc., Chicago, IL, USA).

## 3 RESULTS

### 3.1 Patient Baseline Characteristics

A total of eight patients were included in this study. Baseline characteristics are summarized in Tables **1** and **2**. The median age of the cohort was 44.5 years (range, 30-60 years). The majority of patients exhibited the World Health Organization (WHO) III subtype (n=7, 87.5%). Disease staging revealed one (12.5%), four (50.0%), and three (37.5%) patients with stage II, III, and IVa diseases, respectively. Nodal staging included one (12.5%), three (37.5%), two (25.0%), and two (25.0%) patients with N0, N1, N2, and N3 diseases, respectively. Pre-treatment plasma EBV-DNA was detectable in five patients (62.5%, 95% CI: 30.6-86.3), with a median level of 871 IU/mL (range, 0-697000 IU/mL). The remaining three patients (37.5%) had undetectable plasma EBV-DNA levels prior to treatment.

### 3.2 Treatment before Radiotherapy Interruption Due to the COVID-19 Epidemic

Six patients (75.0%) received IC followed by CCRT, while two (25.0%) underwent CCRT alone. Among the six patients who received IC, one (16.7%) completed two cycles, and five (83.3%) completed three cycles. The median plasma EBV-DNA load post-IC was 0 IU/mL (range, 0-110 IU/mL). Prior to RI due to the COVID-19 pandemic, the median number of IMRT fractions delivered was 10 (range, 4-20 fractions).

### 3.3 Retreatment Following Radiotherapy Interruption

Detailed post-RI treatment characteristics are presented in Table **3**. No patients received additional treatment during RI, and none contracted COVID-19 during this period. All patients returned to our institution to complete IMRT following RI, with a median interruption duration of 19 days (range, 18-25 days). In line with previous studies, one or more additional fractions were an optional strategy to mitigate the adverse effect of RI [26, 27]. All patients received one additional fraction, and six patients (75.0%) received one cycle of CCRT post-RI.

### 3.4 Treatment Response and Compensatory Therapy after the Completion of Radiotherapy

**Fig. (1):**
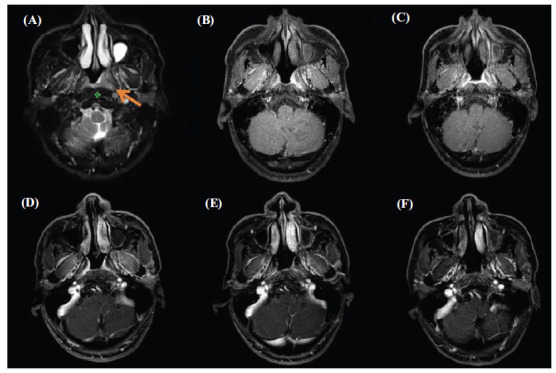
Alterations in the primary nasopharyngeal tumor of patient No. 3 before, during, and after IMRT (**A**) Before induction chemotherapy; (**B**) Before IMRT; (**C**) One month following the completion of IMRT; (**D**) Three months following the completion of IMRT; (**E**) Twelve months following the completion of IMRT; (**F**) Nineteen months following the completion of IMRT) (Orange arrow indicates the primary nasopharyngeal tumor before treatment).

All patients completed the recommended IMRT regimen following RI and underwent comprehensive locoregional and systemic assessment three months post-IMRT. Complete remission (CR) of the nasopharyngeal tumor and cervical lymph nodes was achieved in seven patients (87.5%, 95% CI: 52.9%-97.8%). One patient (12.5%) exhibited partial remission (PR) of cervical lymph nodes but achieved CR of the primary nasopharyngeal tumor (Fig. **1**). The CR rate for the primary nasopharyngeal tumor was 100% (95% CI: 67.6%-100%), while the CR rate for cervical lymph nodes was 85.7% (95% CI: 48.7%-97.3%) (one patient with N0 disease) (Table **4**).

**Fig. (2):**
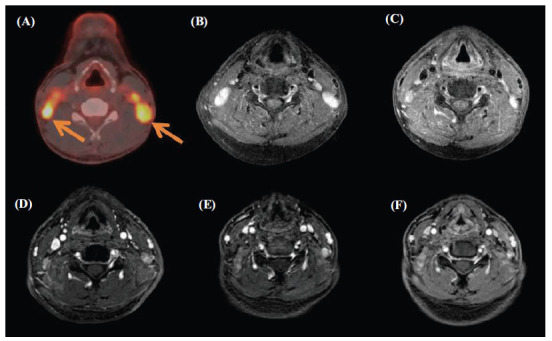
Alterations in the metastatic lymph nodes in the neck of patient No. 3 before, during, and after IMRT (**A**) Before induction chemotherapy; (**B**) Before IMRT; (**C**) One month following the completion of IMRT; (**D**) Three months following the completion of IMRT; (**E**) Twelve months following the completion of IMRT; (**F**) Nineteen months following the completion of IMRT) (Orange arrows indicate the neck metastatic lymph nodes before treatment).

All patients were recommended systemic compensatory therapy, which consisted of oral S-1 MC. All patients completed one year of MC. The patient with PR of cervical lymph nodes continued MC, with the latest assessment confirming CR (Fig. **2**).

### 3.5 Adverse Events During S-1 Treatment

The most common adverse events during S-1 treatment included elevated total bilirubin (n=3, 37.5%), leukopenia (n=2, 25.0%), neutropenia (n=2, 25.0%), anorexia (n=2, 25.0%), rash/desquamation (n=1, 12.5%), and hyperpigmentation (n=1, 12.5%). All adverse events were grade 1-2, with no grade 3-4 toxicities observed.

### 3.6 Survival Outcomes in Patients with Radiotherapy Interruption

The median follow-up duration was 34.4 months (range, 32.7-35.4 months). All patients underwent comprehensive locoregional and systematic evaluation every three months post-IMRT, with no instances of locoregional recurrence or distant metastasis observed. Both the 2-year DFS (95% CI 67.6%-100%) and OS (95% CI 67.6%-100%) were 100%.

## 4 DISCUSSION

The COVID-19 pandemic presented significant challenges to the management of NPC patients, particularly in maintaining the continuity and efficacy of radiotherapy. In this study, we evaluated the treatment characteristics, short-term outcomes, and compensatory treatment using S-1 MC for eight NPC patients who experienced RI at our institution during the COVID-19 pandemic. All patients received at least one year of oral S-1 MC following RI. Our findings suggest that despite RI, treatment outcomes remained favorable, with a high rate of CR and no locoregional recurrences or distant metastases observed during the median follow-up period of 34.4 months.

During the COVID-19 pandemic, approximately 30-50% of NPC patients experienced delays in diagnosis and/or treatment due to city or medical institution lockdowns [17, 28]. Huang *et al*. found that 50% of patients experienced radiotherapy delays during the COVID-19 pandemic. The pandemic introduced unprecedented challenges in managing NPC patients, particularly those undergoing radiotherapy. Our study highlights the complexities of resource reallocation and the difficulties in delivering uninterrupted medical care. Healthcare systems were forced to divert substantial resources to address the surge in COVID-19 cases, often at the expense of timely treatment for non-COVID-19 patients. Ensuring the continuity of care for cancer patients has proven particularly demanding. Moreover, lockdowns, social distancing measures, and travel restrictions significantly hindered patient access to medical services, potentially compromising treatment adherence and follow-up. Nevertheless, our study shows that with meticulous management and the implementation of compensatory therapies, complete remission was achieved in the majority of patients without adversely affecting OS or DFS.

If the initial radiotherapy delay exceeded ≥6 days, 20.4% of patients exhibited elevated EBV-DNA levels, a rate significantly higher than that observed in patients with delays <6 days (3.6%) (*p* <0.001). Furthermore, a radiotherapy delay of ≥6 days was associated with a notable reduction in one-year DFS (91.2% *vs*. 97.8%, *p* =0.006) [17]. In our study, however, EBV-DNA levels remained stable in patients who underwent re-irradiation after RI, and all patients achieved undetectable plasma EBV-DNA levels following the completion of IMRT. This favorable outcome may be attributed to the fact that the majority of our patients received IC, which resulted in undetectable plasma EBV-DNA post-IC. In a recent study, we found that patients with detectable plasma EBV-DNA after IC had significantly worse survival outcomes compared to those with undetectable EBV-DNA levels after IC [29]. Given that the median time to disease recurrence for NPC patients post-IMRT ranges from 24 to 27 months [30, 31], a one-year low-dose MC regimen may help mitigate the risk of recurrence and metastasis in these patients.

The biological mechanisms underlying the compensatory effects of S-1 MC in the context of RI during the COVID-19 pandemic warrant further exploration. Several potential mechanisms have been proposed to explain how S-1 MC may counteract the adverse effects of RI [32-35]. First, MC involves the frequent and continuous administration of low-dose chemotherapeutic agents, which can sustain inhibitory tumor cell proliferation and induce apoptosis, autophagy, and dormancy without the severe toxicities associated with traditional high-dose regimens. This reduced toxicity profile enhances patient tolerance and may improve overall quality of life. Second, S-1 has been shown to activate the immune system, bolstering the body’s antitumor immune responses. This immunomodulatory effect complements the diminished efficacy of interrupted radiotherapy. Third, MC inhibits tumor angiogenesis by downregulating vascular endothelial growth factor expression, thereby reducing the tumor’s blood supply and impeding its growth and metastatic potential. These mechanisms collectively support the clinical application of S-1 MC in NPC patients experiencing RI, offering a promising strategy to optimize treatment outcomes. Additionally, several studies have demonstrated that combining MC with immune checkpoint inhibitors yields higher response rates and improved survival outcomes compared to MC alone (US20150283237) [18, 36-40]. This synergistic approach may further enhance the therapeutic efficacy of MC, particularly in patients with treatment interruptions or advanced disease. Together, these findings underscore the potential of S-1 MC as a valuable compensatory strategy in the management of NPC patients facing RI or radiotherapy delays, ultimately contributing to better clinical outcomes and patient care.

The inclusion of patent references underscores the innovative nature of MC in NPC (US20150283237, CN111110681A) [18, 19]. These patents highlight advancements in drug delivery systems and pharmacokinetic optimization, which enhance the efficacy and tolerability of MC. For instance, patents focusing on sustained-release formulations of S-1 have shown potential in maintaining therapeutic drug levels while minimizing toxicity. The application of these innovations in our study likely contributed to the favorable outcomes observed. Future research should explore the integration of these patented technologies into broader clinical practice, particularly in resource-limited settings or during healthcare disruptions like those caused by COVID-19.

RI can occur due to various factors, including public holidays, machine malfunctions, acute treatment-related toxicities, and patient non-compliance. Several studies have found that RI lasting ≥4-7 days is associated with an increased risk of locoregional and distant failure [7, 8, 41]. In China, public holidays such as the National Day and Spring Festival typically span approximately 7 days. To mitigate the adverse effects of RI on patient outcomes, our institution implements a strategy of administering 3 to 4 radiotherapy sessions during these extended holidays, ensuring treatment continuity. Notably, Xu *et al*. found that escalating the cisplatin dosage during CCRT could compensate for the detrimental effects of RI in NPC patients [6]. The COVID-19 pandemic posed unique challenges, as outpatient radiotherapy was frequently interrupted due to medical institutions or city-wide lockdowns. In our cohort, all patients resumed radiotherapy at our institution once pandemic-related restrictions were temporarily lifted. Ying *et al.* reported that 72.1% of patients returned for further radiotherapy after a median RI duration of 15 days [15]. In our study, the median RI duration was 19 days, and all patients received an additional fraction of IMRT upon resumption. This approach ensured the delivery of the prescribed radiation dose, thereby preserving therapeutic efficacy.

A study from Tongji Hospital in Wuhan City documented 14 NPC patients who experienced RI during the city’s lockdown, with 13 patients returning for subsequent radiotherapy. During follow-up, two patients developed liver metastasis at 4.6 and 1.3 months post-radiotherapy, with RI durations of 75 and 103 days, respectively [15]. However, details regarding compensatory treatments for these patients were not recorded. In our study, with a median follow-up of 34.4 months, all patients received at least one year of oral S-1 MC, and no disease recurrence was observed. One patient with an RI duration of 25 days exhibited persistent bilateral cervical lymph node enlargement three months post-IMRT. This patient continued oral S-1 MC, resulting in gradual lymph node regression, undetectable plasma EBV DNA, and no evidence of disease progression on regular systemic evaluations. To our knowledge, this is the first study to explore compensatory treatment strategies for NPC patients experiencing RI during the COVID-19 pandemic.

MC, characterized by the sustained administration of low-dose chemotherapy agents, has been shown to induce tumor cell dormancy, inhibit angiogenesis, modulate the immune system, and exert antitumor effects [42, 43]. Prospective studies have demonstrated improved survival outcomes with MC in various cancers, including breast cancer and head and neck cancer [44, 45]. Retrospective and prospective studies have also found that one year of capecitabine, tegafur uracil, or S-1 MC significantly enhances failure-free survival and OS in LANPC [25, 46-48]. While several studies have highlighted poorer survival outcomes in patients with RI [7, 8], compensatory treatment strategies for this population remain underexplored. Notably, two prospective studies concluded that adjuvant chemotherapy does not improve survival in LANPC and may increase treatment-related toxicity [49, 50]. Our findings suggest that MC represents an effective and low-toxicity compensatory strategy for patients experiencing RI. The implications of this study are significant for clinical practice, particularly in the context of pandemics or other unforeseen interruptions to radiotherapy. The successful management of RI through timely treatment resumption and compensatory therapies underscores the importance of a flexible and patient-centered approach. This strategy not only ensures the delivery of essential therapeutic regimens but also maintains high CR rates and favorable long-term outcomes. However, further validation through prospective studies with large sample sizes is warranted to confirm these findings.

According to previous studies, administering one or more additional fractions has emerged as an optional strategy to mitigate the adverse effects of RI [26, 27]. In our study, all patients received an extra fraction due to RI. However, alternative approaches such as scheduling treatments on weekends, developing a larger number of remaining fractions, or using a twice-daily fractionation regimen might be preferable for compensating for the negative impacts of RI [27, 51]. In our study, patients were prescribed a single fraction dose of 2.13 Gy for the gross tumor volume, which aligns with the hypofractionation regimen. For patients with NPC, either increasing the dose per fraction or implementing twice-daily fractionation according to the current protocol could potentially elevate the incidence of early and late normal tissue reactions [52]. In those with head and neck cancers, scheduling radiotherapy on the weekend was associated with higher acute morbidity, especially the confluent mucositis and severe skin reactions [53, 54]. In addition, the use of twice-daily accelerated fractionation was also associated with a higher risk of acute and late toxicity in head and neck cancer compared to those using conventional fractionation [55, 56]. Therefore, our patients predominantly employed a compensation strategy of adding one extra fraction of the original radiation dose. The RI period induced by hospital lockdowns during the COVID-19 pandemic could be notably prolonged. In this study, the median duration of RI among patients reached 19 days. Therefore, after patients completed the recommended radiotherapy dose, we administered an additional fraction of radiotherapy followed by MC as a compensatory treatment, aiming to minimize the adverse effects of RI to the greatest extent possible. However, given the lack of recommended compensatory treatments for such a long-term RI at present, we are unable to compare the differences in survival outcomes of patients across other potential compensatory treatment modalities.

## STUDY LIMITATIONS

Several limitations should be acknowledged in our study. Firstly, as with all retrospective studies, inherent biases are inevitable. Patient selection and treatment decisions can be influenced by a multitude of factors, including patient condition and physician preference. However, the RI caused by unexpected events has provided us with some therapeutic insights. Moreover, designing a randomized controlled trial for RI in NPC poses challenging ethical considerations in practice. Secondly, the small sample size of patients and the short follow-up period limit the generalizability of our study findings to the broader population of NPC patients with RI. Despite these limitations, the clinical relevance of our findings is significant. The 100% 2-year DFS and OS rates suggest that oral MC with S-1 is a viable compensatory strategy for NPC patients with RI. This approach may be particularly valuable in scenarios where conventional treatments are disrupted. A larger sample size study with long-term follow-up is required to determine the role of MC in NPC patients experiencing RI. In addition, the absence of a control group prevents meaningful comparisons with standard management approaches, making it difficult to determine whether MC directly influenced the observed survival rates. Finally, current guidelines suggest that patients infected with COVID-19 should postpone radiotherapy for at least 10 days [57]. However, none of the patients in our study had a COVID-19 infection. Therefore, further research is required to explore the role of MC in NPC patients with both COVID-19 infection and RI.

## CONCLUSION

The COVID-19 pandemic has had a substantial impact on the continuity and efficacy of radiotherapy. Our study suggests that S-1 MC may serve as an effective compensatory treatment approach, leading to favorable survival outcomes for NPC patients experiencing RI. This study not only addresses the management challenges posed by the COVID-19 pandemic but also provides valuable insights for future clinical trials involving NPC patients with RI caused by various reasons. The novel application of MC in this scenario represents a significant practical contribution to improving patient outcomes and guaranteeing the continuity of medical care. However, these findings are exploratory in nature and necessitate validation through a larger-scale, prospective, controlled study.

## Figures and Tables

**Table 1:** Patient baseline characteristics.

**Variables**	**n (%)**
**Gender**		
Male	5 (62.5)
Female	3 (37.5)
**Age**		
<50 years	5 (62.5)
≥50 years	3 (37.5)
**Smoking History**		
No	4 (50.0)
Yes	4 (50.0)
**Alcohol Consumption**		
No	6 (75.0)
Yes	2 (25.0)
**Clinical Stage**			
I	0 (0)
II	1 (12.5)
III	4 (50.0)
IVA	3 (37.5)
**EBV-DNA Level Before Treatment (IU/mL)**			
<2000	6 (75.0)
≥2000	2 (25.0)
**Induction Chemotherapy Regimen**			
No	2 (25.0)
Gemcitabine-based	3 (37.5)
Taxane-based	3 (37.5)

**Table 2:** Details of patient characteristics before IMRT.

**Patient No.**	**Gender**	**Age (Years)**	**Time for NPC ** **Diagnosis**	**Histology**	**Stage**	**Induction ** **Chemotherapy**	**EBV-DNA Load Before Treatment (IU/mL)**	**EBV-DNA Load After IC (IU/mL)**
1	Male	36	May 25, 2021	WHO III	T2N2M0	Yes	8680	0
2	Male	38	May 28, 2021	WHO III	T2N2M0	Yes	512	0
3	Male	44	June 2, 2021	WHO III	T2N3M0	Yes	1900	0
4	Male	59	June 8, 2021	WHO III	T2N3M0	Yes	1230	110
5	Female	45	August 13, 2021	WHO III	T3N0M0	No	0	NA
6	Female	50	July 10, 2021	WHO III	T2N1M0	Yes	0	0
7	Male	63	July 15, 2021	WHO II	T4N1M0	Yes	697000	0
8	Female	30	July 28, 2021	WHO III	T3N1M0	No	0	NA

**Table 3:** Patient characteristics during and after IMRT.

**Patient No.**	**Date of First IMRT Treatment**	**CCRT**	**Number of Fractions When RI**	**Days of RI**	**Number of Additional Fractions**	**EBV-DNA Load After Three Months of IMRT (IU/mL)**	**Metronomic Chemotherapy**
1	August 30, 2021	Yes	10	18	1	0	Yes
2	August 16, 2021	Yes	20	18	1	0	Yes
3	August 19, 2021	Yes	17	25	1	0	Yes
4	August 23, 2021	Yes	15	22	1	0	Yes
5	September 7, 2021	Yes	4	19	1	0	Yes
6	September 7, 2021	Yes	4	18	1	0	Yes
7	September 2, 2021	Yes	7	19	1	0	Yes
8	August 30, 2021	Yes	10	20	1	0	Yes

**Table 4:** Treatment response three months after the completion of IMRT.

**Treatment Response**	**Entire Cohort (%)**	**Nasopharyngeal Tumor (%)**	Cervical Lymph Nodes (%) *
CR	7 (87.5)	8 (100)	6 (85.7)
PR	1 (12.5)	0 (0)	1 (14.3)

**Note:** *Indicates 7 out of these 8 patients had cervical lymph node-positive disease.

## Data Availability

The data supporting the findings of the article are available within the article.

## LIST OF ABBREVIATIONS

CCRT: Concurrent Chemoradiotherapy
CI: Confidential Interval
COVID-19: Coronavirus Disease 2019
CR: Complete Remission
CTCAE: Common Terminology Criteria for Adverse Events
DFS: Disease-free Survival
EBV: Epstein-Barr Virus
Gy: Gray
IC: Induction Chemotherapy
IMRT: Intensity-modulated Radiation Therapy
LANPC: Locoregionally Advanced Nasopharyngeal Carcinoma
MC: Metronomic Chemotherapy
MR: Magnetic Resonance
MTD: Maximum Tolerated Dose
N: Nodal
NPC: Nasopharyngeal Carcinoma
OS: Overall Survival
PET/CT: Positron Emission Tomography/Computed Tomography
PR: Partial Remission
RECIST: Response Evaluation Criteria for Solid Tumors criteria
RI: Radiotherapy Interruption
S-1: Tegafur, Gimeracil and Oteracil Potassium
WHO: World Health Organization
